# The Pro-Oxidant Activity of Red Wine Polyphenols Induces an Adaptive Antioxidant Response in Human Erythrocytes

**DOI:** 10.3390/antiox10050800

**Published:** 2021-05-18

**Authors:** Idolo Tedesco, Carmela Spagnuolo, Gian Luigi Russo, Maria Russo, Carmen Cervellera, Stefania Moccia

**Affiliations:** National Research Council, Institute of Food Sciences, 83100 Avellino, Italy; idolo.tedesco@isa.cnr.it (I.T.); carmela.spagnuolo@isa.cnr.it (C.S.); glrusso@isa.cnr.it (G.L.R.); mrusso@isa.cnr.it (M.R.); carmen.cervellera@isa.cnr.it (C.C.)

**Keywords:** red wine polyphenols, PMRS, erythrocytes, antioxidant, adaptive response

## Abstract

The protective effect of dealcoholized red wine on human health has been partially associated with its polyphenolic components, suggesting that the pool of polyphenols, including flavonoids and anthocyanins, can be responsible for the functional effects of this beverage. We hypothesize a new role of red wine polyphenols (RWp) in modulating the antioxidant potential of erythrocytes, protecting them against oxidative stress. We previously demonstrated that RWp activated the Plasma Membrane Redox System (PMRS), which is involved in neutralizing plasma free radicals. Here, we investigated the underlying mechanism triggered by RWp in the activation of PMRS via the involvement of GSH. Hence, treatment of human erythrocytes with RWp (73 μg/mL Gallic Acid Equivalents) increased GSH intracellular concentration, which depends upon the activation of glutathione reductase (GR) and glucose-6-phosphate dehydrogenase (G6PD), whose enzymatic activities increase of about 30% and 47%, respectively. Changes in the GSH pathway induced by RWp were associated with a slight but significant increase in reactive oxygen species (ROS). We conclude that the pro-oxidant effect of RWp promoted an adaptive stress response in human erythrocytes, which enhances their antioxidant defense.

## 1. Introduction

Over the years, several studies have strengthened the role of diet in the prevention of chronic diseases, highlighting how dietary intervention can promote health status and restore imbalances induced by lifestyle and environmental factors [1,2]. Among these, changes in dietary patterns including moderate wine consumption could have a positive role in health promotion and disease risk prevention, like cardiovascular and metabolic syndrome, neuronal cognitive decline, and cancer [3,4,5]. 

The health-promoting effect of red wine (RW) had been supported by epidemiological evidence, indicating that RW components could improve endothelial dysfunction and hypertension, dyslipidemia, and metabolic disorders [6]. The beneficial role of RW consumption in reducing the incidence of metabolic syndrome was also supported by the PREDIMED (Prevención con Dieta Mediterránea) study in elderly Mediterranean people at high cardiovascular risk [7,8]. Another interesting evidence suggested the involvement of RW in the modulation of gut microbiota, contributing to the microbial balance resulting in an improvement in health status [9]. However, despite these in vivo promising results, more randomized clinical trials are necessary to better define the involvement of RW in reducing the related risk of several pathological conditions.

The positive role of RW on human health has been bona fide attributed to its phytochemical compounds, including polyphenols, as suggested by several clinical trials [10,11]. Among polyphenols, it should be considered that RW polyphenolic composition is complex and variable, and can be influenced by the enzymatic and non-enzymatic reactions, which occur in the winemaking processes throughout the fermentation process and wine aging [12]. Red wine polyphenols (RWp) can be divided into two main categories: non-flavonoid compounds, such as phenolic acids (hydroxybenzoic and hydroxycinnamic acids), stilbenes, and flavonoid compounds, including flavones, flavonols, flavanones, flavanols, and anthocyanins [13]. Anthocyanins include monomers, such as (+)-catechin and (−)-epicatechin, and more complex oligomeric structures or polymers called proanthocyanidins [13]. These last represent the largest group of polyphenols in RW (60–80%) possessing very complex structures, which mainly include polymeric procyanidins and prodelphinidins. They are formed by molecules such as epicatechin, catechin, epicatechin-3-*O*-gallate that are linked to acetaldehyde through 1,1-ethylene bridge [14,15]. The biological role of RW polymers (RWplm) remains largely unknown, although they have shown in vivo and in vitro antioxidant activity, which could also contribute to the effect of RWp against oxidative stress [16,17]. 

As reported above, the biological properties of RW can be partially explained by the presence of phenolic compounds able to interact with physiological targets, through different mechanisms [18]. Considering the cellular models, the potential cardioprotective activity of RWp has been mainly linked to interactions with endothelial functions, promotion of vascular homeostasis through the release of nitric oxide (NO), and inhibition of platelet aggregation [19,20]. For example, it has been demonstrated that RW anthocyanins protected strongly against H_2_O_2_-generated hemolysis in human erythrocytes (normal and catalase-inactive) [21]. It is supposed that hydroxyl groups and the system of conjugated double bonds could be responsible for this effect, but we cannot exclude that their biological effects could also be related to other mechanisms [22]. For example, it has been reported that RW anthocyanin derivatives can interact with the lipid bilayer membrane explaining their capacity to reduce lipid peroxidation [23]. It is also possible that the cellular antioxidant systems are triggered by the oxidation of the hydroxyl groups of the phenolic rings of RWp, which is mediated by polyphenol oxidase and leads to the formation of radical intermediates [24].

The modulation of antioxidant endogenous enzymes was also considered as one of the possible mechanisms by which RWp could exert its protective role as reported by in vivo and in vitro studies [25,26]. The role of exogenous antioxidants in the modulation of the cellular antioxidant system has been considered an important strategy to improve erythrocyte defense mechanisms [18]. For example, it has been reported that subjects receiving a diet poor in antioxidants showed reduced enzymatic activity, including catalase (CAT), glutathione peroxidase (GPx), and glutathione reductase (GR) [25].

Among the antioxidant systems, we previously demonstrated that RWp activated erythrocytes Plasma Membrane Redox System (PMRS) [26], a transmembrane enzymatic complex that plays a crucial role in the regulation of cellular homeostasis and redox state, aging, lifespan, and several oxidative stress-induced pathological conditions [27,28,29]. 

PMRS is ubiquitously present in all cells and it is composed of oxidoreductase enzymes, such as NAD(P)H-dependent redox enzymes, NADH-ferricyanide reductase, cytochrome b5 reductase, and NAD(P)H-quinone oxidoreductase 1 (NQO1). PMRS can neutralize external oxidative species through the transfer of electrons across the cell membrane from intracellular compounds that are capable of donating electrons, such as from NADH or ascorbate (ASC), reduced glutathione (GSH), to extracellular electron acceptors, such as ascorbyl free radical (AFR) [30]. PMRS is involved in the maintenance of a balanced NAD(P)/NAD(P)H ratio, essential for cellular functions and in recycling ASC, through the reduction of AFR to avoid the formation of dehydroascorbate (DHA), the oxidized form of ASC [31]. ASC reducing power plays an important role in maintaining the redox state due to its ability to donate electrons, preserving their intracellular levels in the millimolar range through different mechanisms of recycling [32]. Another important factor involved in the regulating mechanisms of the intracellular redox state is GSH, which also modulates the PMRS activity, as we confirmed previously [26].

In the present work, we investigate the mechanism triggered by RWp in protecting human erythrocytes from oxidative stress throughout the activation of PMRS and we suggest a new role of RWp in regulating antioxidant defense. We also examined the contribution of RWplm in proposing how these compounds can contribute to the protective role of RWp.

## 2. Materials and Methods

### 2.1. Chemicals

Phosphate buffer saline (PBS) tablets were from Life Technologies (Monza, Italy); hydrochloric acid (HCl); sodium acetate (NaOAc); citric acid; glucose, ethylenediaminetetraacetic acid (EDTA), Tris (hydroxymethyl) aminomethane hydrochloride (Tris-HCl) were from Carlo Erba (Milan, Italy). Lithium sulphate; 2-mercaptoethanol; DL-glyceraldehyde; gallic acid; ferrous sulfate (FeSO_4_); potassium ferricyanide (K_3_Fe(CN)_6_); ferric chloride (FeCl_3_); bathophenanthrolinedisulfonic acid disodium salt hydrate; trichloroacetic acid (TCA); glutathione (GSH); phtaldialdehyde; Folin–Ciocalteau’s reagent (FCR); 2,4,6-Tris (2-pyridyl)-triazine (TPTZ); dichlorofluorescein-diacetate (DCFDA); nicotinamide adenine dinucleotide phosphate reduced (NADPH); nicotinamide adenine dinucleotide reduced (NADH); phenylmethylsulfonyl fluoride (PMSF); flavin adenine dinucleotide (FAD); oxidized glutathione (GSSG); magnesium chloride (MgCl_2_); pyrogallol; glucose-6-phosphate (G6P); xylenol orange; ammonium ferrous sulfate; butylated hydroxytoluene (BHT); sodium azide were from Merck (Milan, Italy). All other chemicals used were of research highest purity grade.

### 2.2. Preparation of Erythrocytes

Peripheral blood samples from ten healthy donors were provided by the Blood Donation Centre at the Onco-Hematology Department of “San Giuseppe Moscati” Hospital (Avellino, Italy) with informed consent. The participants (*n* = 10) were healthy donors, not-smokers, without comorbidities. They included 6 males and 4 females with an average age of 41 and 38 years, respectively. After the collection of whole blood samples in EDTA-treated tubes, the erythrocytes were separated by centrifugation and treated with PBS to remove plasma, platelets, and buffy coat.

### 2.3. Preparation of Red Wine Polyphenols (RWp) and Polymers

An experimental RW made from “Aglianico” grapes and obtained by a microvinification process was employed [26]. RWp content was measured by Folin–Ciocalteu’s assay and quantified as μg/mL gallic acid equivalent (GAE), a polyphenol present in significant amounts in RW [33]. The separation of RWplm with high molecular weight was performed through dialysis of RW against 0.01 N HCl using a membrane of 3000 kDa cut-off (Merck) under stirring for 72 h [34]. For the cellular tests, after drying, RWp and RWplm were solubilized in 0.01 N HCl. Green tea (Lipton® Uniliver U.K.) and karkadè (Pompadour® Bolzano, Italy) were purchased from a local supplier and single bags were infused for 15 min in 20 mL and 10 mL of hot water (80 °C), respectively. After cooling, aliquots of the solutions were centrifuged at 1800× *g* for 5 min, and immediately used for dialysis. Karkadè polymers (Kplm) and green tea polymers (Tplm) were obtained with the same procedure reported above for RWplm and both were suspended in 0.01 N HCl.

### 2.4. Measurement of Reactive Oxygen Species (ROS)

The measurement of ROS intracellular concentration was performed using DCFDA, a non-fluorescent molecule that crosses the cellular membrane and is hydrolyzed to dichlorofluorescein. The latter is converted by intracellular peroxides into DCF, which can be measured by spectrofluorimetry. Briefly, erythrocytes (8 × 10^5^ erythrocytes/μL) were treated for 30 min with 20 μM DCFDA and after several washes in PBS, were stimulated with RWp (73 μg/mL GAE) at different times (1 and 10 min). The production of ROS was determined by fluorimetry with excitation and emission settings at 495 and 530 nm, respectively.

### 2.5. ErythrocytesPreparation for Antioxidant Enzyme Measurement

After isolation and wash with PBS, erythrocytes (8 × 10^5^ erythrocytes/μL) were incubated with RWp (73 μg/mL GAE) at different times (1–10 min). At the end of incubation, the samples were centrifuged at 1800× *g*, washed with PBS, and finally lysed using 5 mM phosphate buffer, pH 8.0 containing 1 mM PMSF. The samples were centrifuged at 11000× *g* and supernatants were collected for the enzymatic assays.

#### 2.5.1. Glutathione Reductase (GR) Activity

GR activity was determined according to Lopez et al. [35]. Samples were treated for 5 min at 37 °C with 5.1 μM FAD, 0.16 mM NADPH, 0.49 mM EDTA. To measure the oxidation of NADPH to NADP^+^, which occurs through the GSSG reduction, 1.95 mM GSSG was added to start the reaction. The absorbance was measured at 340 nm after 30 min and the specific enzymatic activity was expressed as nmol/min/mL erythrocytes.

#### 2.5.2. Glucose-6-Phosphate Dehydrogenase (G6PD) Activity

G6PD activity was determined according to Akkemik et al. [36] by measuring the production of NADPH at 340 nm. Samples were treated for 5 min at 37 °C in the presence of 0.3 mM NADP, 10 mM MgCl_2_ in 100 mM Tris-HCl, pH 8.0. After incubation, 1 mM G6P was added to start the reaction. The enzymatic activity was expressed as nmol/min/mL erythrocytes.

#### 2.5.3. Superoxide Dismutase (SOD) Activity

SOD activity was determined as previously reported by Li [37]. Samples were treated for 5 min at 37 °C in a buffer containing 50 mM Tris-HCl, pH 7.4, 1 mM EDTA. After incubation, 50 mM pyrogallol was added to start the reaction at 37 °C. The absorbance was determined after 20 min and the specific enzymatic activity was expressed as U/mL erythrocytes.

#### 2.5.4. Catalase (CAT) Activity

CAT activity was measured according to Mendiratta et al. with some modifications [38]. Briefly, samples were incubated in the presence or absence of 5 mM sodium azide for 10 min before the addition of 0.5 mM H_2_O_2_ for 3 min, and incubated for 30 min in the presence of FOX reagent (100 mM xylenol orange, 250 mM ferrous ammonium sulfate, and 100 mM sorbitol in 25 mM H_2_SO_4_). The absorbance was measured at 560 nm and the enzymatic activity was expressed as percentage of inhibition.

#### 2.5.5. Aldose Reductase (ALR) Activity

Aldose reductase was determined according to Akileshwari et al. with some modifications [39]. Briefly, samples were incubated with 0.4 mM Li_2_SO_4_, 6 mM 2-mercaptoethanol, and 0.24 mM NADPH in 0.1 M sodium phosphate buffer pH 6.2. After incubation at 37 °C for 5 min, the reaction was started with the addition of 2.5 mM DL-glyceraldehyde for 30 min. The absorbance was measured at 340 nm, and the specific activity was reported as nmol/min/mL erythrocytes.

#### 2.5.6. NADH-Methaemoglobin Reductase (MetHbR) Activity

MetHbR was measured according to Board with some modifications [40]. Samples were incubated with 4 mM EDTA and 1.76 mM NADH in water. After incubation at 37 °C for 5 min, the reaction was started with the addition of 0.2 mM K_3_Fe(CN)_6_ for 30 min. The absorbance was measured at 340 nm and the specific activity reported as nmol/min/mL erythrocytes. 

### 2.6. Determination of Plasma Membrane Redox System (PMRS)

To measure PMRS activity, erythrocytes (8 × 10^5^ erythrocytes/μL) were diluted with PBS and incubated at 37 °C for 10 min using volumes of RWp and RWplm corresponding to 73 μg/mL GAE and 27 μg/mL GAE, respectively. Alternatively, erythrocytes were incubated with different volumes corresponding to a final concentration of 73 μg/mL GAE for RWp and RWplm. Erythrocytes were washed with PBS and treated with a mixture containing PBS, 5 mM glucose, and 1 mM K_3_Fe(CN)_6_ at 37 °C for 30 min. After centrifugation at 1800 × *g*, the supernatants were collected for PMRS assay as previously reported [26]. Absorbance was measured at 540 nm and results were expressed as picomoles ferrocyanide/10^6^ erythrocytes/min. 

### 2.7. Measurement of GSH 

Erythrocytes (8 × 10^5^ erythrocytes/μL) were diluted with PBS and incubated at 37 °C for 10 min with final volumes of RWp and RWplm corresponding to 73 μg/mL GAE and 27 μg/mL GAE, respectively. Samples were washed with PBS, and solubilized with TCA solution (5% *v*/*v* in 0.1 M HCl, 10 mM EDTA). Samples were treated with phtaldialdehyde (0.5 mg/mL) and 10 mM EDTA. The fluorescence of supernatants was measured at 340 nm (excitation wavelength) and 460 nm (emission wavelength) [26]. The micromolar concentration of GSH was calculated from a standard curve of pure GSH.

### 2.8. Statistical Analysis

Data are presented as mean values ± standard error (SE) and the significance was measured by the use of Student’s test for the evaluation of the single treatment vs the average of the controls. The significance level was fixed at 0.05 for all the statistical analyses; values with *p* < 0.05 were considered statistically significant.

## 3. Results

We firstly evaluated the total phenolic content of an experimental “Aglianico” RW, resulting in 2190 ± 0.05 μg/mL GAE, a value falling in the range of other red wines [26]. We collected by dialysis RWplm and measured the total polyphenol amount (810 ± 0.03 μg/mL GAE), which represented about 37% of the total polyphenol content present in RW.

### 3.1. Red Wine Pro-Oxidant Effect in Erythrocytes 

We previously demonstrated the involvement of RWp in modulating erythrocytes’ antioxidant system by activation of PMRS, which represents one of the key defense mechanisms of erythrocytes against oxidative stress and hemolysis [26]. We also showed that this mechanism was mediated by the increase of GSH intracellular concentration, since its crucial role as an intracellular electron donor to PMRS [26]. We summarized our data previously published [26] in Table 1, reporting the protection from hemolysis, the activation of PMRS, and the increase of GSH on erythrocytes by RWp (73 μg/mL GAE).

Here, we hypothesized that RWp could interfere with intracellular ROS concentration, which, in turn, mediates the GSH response. Erythrocytes were incubated with the same concentration of RWp (73 μg/mL GAE) used previously and responsible for the biological activities reported in Table 1 [26]. This concentration was selected in the range of those reported in previous work that measured the circulating concentration of phenolic compounds in human plasma after regular consumption of RW, e.g. 375 mL/day for two weeks [41]. In addition, the short times of incubation are consistent with those previously reported [26] and are justified by the expected rapid modulation of ROS production and metabolism observed in biological systems [42]. 

**Figure 1:**
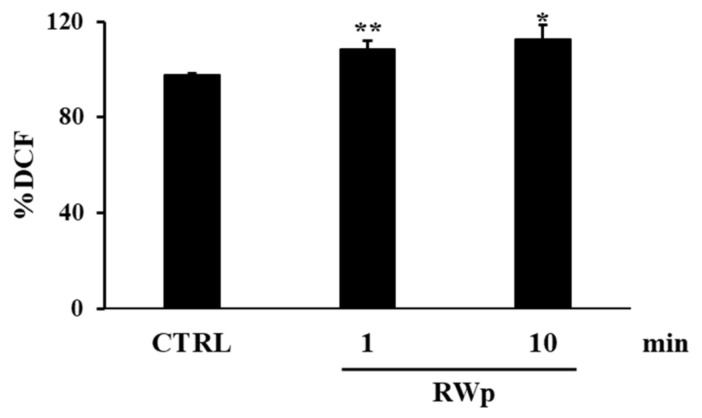
ROS production induced by RWp. Erythrocytes isolated from volunteers (*n* = 5) were incubated with RWp (73 μg/mL GAE) at the indicated times. Intracellular ROS levels were determined by using DCFDA/DCF fluorescent method and the results were expressed as % DCF, as described in “Materials and Methods”. Bar graphs represent the mean of 5 independent determinations, each performed in duplicate, ± SE. Symbols (*) in the graph indicate statistical significance: * *p* < 0.05 and ** *p* < 0.01 respect to untreated cells (CTRL).

For this purpose, we measured ROS production following RWp treatment and, as reported in Figure 1, we detected a significant increase in intracellular ROS concentration of 108 ± 3.2 and 112 ± 5.8 (% DCF) after 1 min and 10 min incubation, respectively, compared to 97 ± 0.4 (% DCF) of untreated erythrocytes (CTRL).

To evaluate whether the pro-oxidant activity of RWp was associated with the production of toxic aldehydes or the oxidation of ferrous to ferric ions of the hemoglobin, we measured the enzymatic activities of ALR and MetHbR, respectively. Data reported in Table 2 indicate that RWp (73 μg/mL GAE) up to 10 min did not induce any toxic product, suggesting that the pro-oxidative mechanism of RWp was not associated with any harmful effect on erythrocytes.

ALR and MetHbR activities were expressed as nmol/min/mL erythrocytes. No significant difference was reported between treatment at 10 min and untreated cells (CTRL). All data are the mean of 5 independent determinations, each performed in duplicate, ± SE.

### 3.2. Activation of Anti-Oxidant Enzymes by RWp

**Figure 2:**
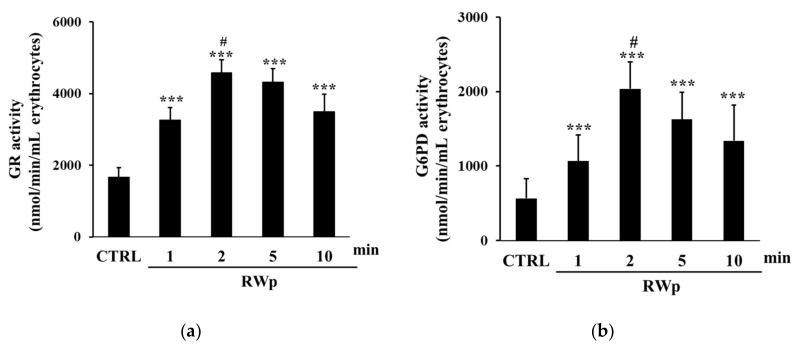
Activation of GSH-dependent enzymes by RWp. Erythrocytes from volunteers (*n* = 7) were incubated with RWp (73 μg/mL GAE) at the indicated times. (**a**) GR activity of erythrocytes, expressed as nmol/min/mL erythrocytes. (**b**) G6PD activity of erythrocytes, expressed as nmol/min/mL erythrocytes. Bar graphs represent the mean of 7 independent determinations, each performed in duplicate, ± SE. Symbols (*) in the graph indicate statistical significance: *** *p* < 0.001 respect to untreated cells (CTRL); # *p* < 0.05 significance between 1 and 2 min of incubation with RWp.

GSH represents the first line of cellular defense to counter the increase in ROS production, scavenging free radicals or acting as substrate electron donors to PMRS [43]. We previously demonstrated that RWp (73 μg/mL GAE) significantly increased GSH intracellular concentration in human erythrocytes [26]. To demonstrate whether the increase of GSH was due to the activation of GR, its activity, which participates in restoring the intracellular GSSH/GSSG ratio, was measured. The possibility that the conversion from GSSG to GSH was associated with the increase in GR enzymatic activity was also supported by the unlikely de novo synthesis of GSH due to its rapid increase [26]. As reported in Figure 2a, RWp (73 μg/mL GAE) significantly increased GR activity of about 1.7-fold after 2 min of treatment compared to untreated erythrocytes. Moreover, it has been also observed a significant increase of G6PD activity, detected after 2, 5, and 10 min of treatment with RWp (73 μg/mL GAE), peaking at 2 min with about a 3.6-fold increase (Figure 2b). These data suggest that GR enzyme is responsible for the depletion of its cofactor, NADPH, whose intracellular pool was restored by G6PD, the enzyme required to produce NADPH in the pentose phosphate pathway and that protects erythrocytes from oxidative stress [44]. 

**Figure 3:**
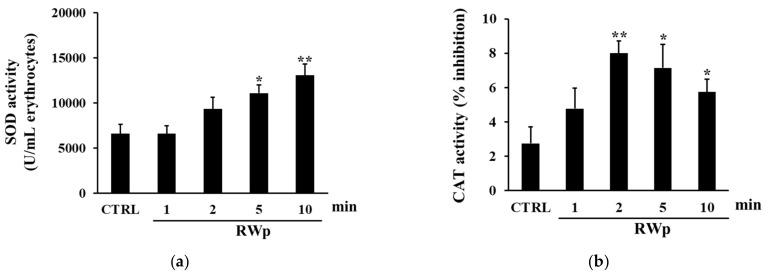
Activation of antioxidant enzymes by RWp. Erythrocytes from volunteers (*n* = 5) were incubated with RWp (73 μg/mL GAE) at the indicated times. (**a**) SOD activity of erythrocytes, expressed as U/mL erythrocytes. (**b**) CAT activity of erythrocytes, expressed as % inhibition. Bar graphs represent the mean of 5 independent determinations, each performed in duplicate, ± SE. Symbols (*) in the graph indicate statistical significance: * *p* < 0.05 and ** *p* < 0.01 respect to untreated cells (CTRL).

To verify the activation of key antioxidant enzymes involved in the defense against the harmful effects of ROS, SOD, and CAT activities were measured. SOD acts in almost all cell types exposed to oxygen by catalyzing the dismutation reaction of the superoxide radical into molecular oxygen or hydrogen peroxide [44]. As reported in Figure 3a, when we measured the kinetic of SOD activation, it increased significantly in erythrocytes treated with RWp (73 μg/mL GAE) after 5 and 10 min, compared to control. These data are reinforced by the observation that RWp induced a superoxide anions production in erythrocytes, which could be responsible for the activation of SOD (data not shown). Therefore, the degradation of hydrogen peroxide to water and molecular oxygen was catalyzed by CAT, which is responsible for the control of hydrogen peroxide levels. As shown in Figure 3b the treatment with RWp (73 μg/mL GAE) was able to significantly increase CAT activity compared to untreated controls. 

The intracellular peroxide levels are also maintained by the activity of glutathione peroxidase (GPx), a GSH-dependent enzyme that has an important function in cellular antioxidant protection. Unexpectedly, we did not measure changes in the GPx enzymatic activity after treatment with RWp (data not shown).

### 3.3. Role of RWplm in modulating PMRS activity

To investigate the potential contribution of high molecular weight RWp components, we separated RWplm from whole RW by dialysis and tested this fraction on erythrocytes. Firstly, we evaluated the PMRS activity after the treatment with the same volume of RWp and RWplm (corresponding to 73 and 27 μg/mL GAE, respectively). The concentration of 27 μg/mL GAE, used for RWplm, corresponded to the amount of polymers present in 73 μg/mL GAE of total RWp.

**Figure 4:**
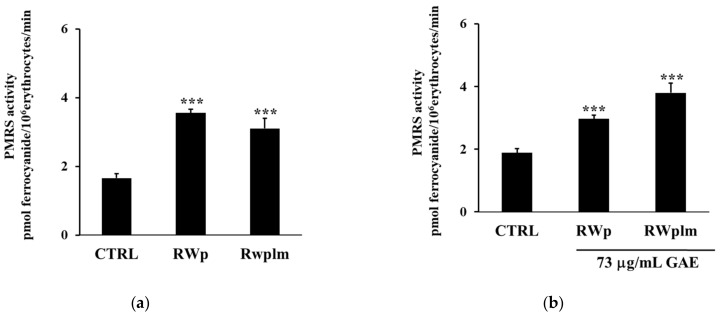
Activation of PMRS by RWplm. Erythrocytes from volunteers (*n* = 5) were incubated in presence or absence of RWp and RWplm. (**a**) PMRS activity of erythrocytes incubated for 10 min with RWp and RWplm (73 μg/mL GAE, 27 μg/mL GAE, respectively). (**b**) PMRS activity of erythrocytes incubated for 10 min with the same concentration of RWp and RWplm (73 μg/mL GAE). PMRS activity was expressed as pmol ferrocyanide/10^6^ erythrocytes/min as reported in “Materials and Methods”. Bar graphs represent the mean of 5 independent determinations, each performed in duplicate, ± SE. Symbols (*) in the graph indicate statistical significance: *** *p* < 0.001 respect to untreated cells (CTRL).

The incubation with RWp highlighted a significant increase in PMRS activity compared to the untreated control (Figure 4a). It is interesting to note that the difference in the PMRS activity between the two treatments, RWp and RWplm, reported in Figure 4a, was not significant, suggesting that both these preparations increased the activity of PMRS to the same extent. Therefore, we repeated the assay using the same concentration (73 μg/mL GAE) of RWp and RWplm. As expected, RWplm was found to be more effective in improving PMRS activity with respect to RWp (Figure 4b).

To evaluate if the effect of RWplm was not-specific and simply due to the bulky structure of the polymers, we tested the polymeric fraction prepared from other beverages, which contain comparable amounts of polymers including tea (*Camellia sinensis* L.) and karkadè (*Hibiscus sabdariffa* L.). 

**Figure 5:**
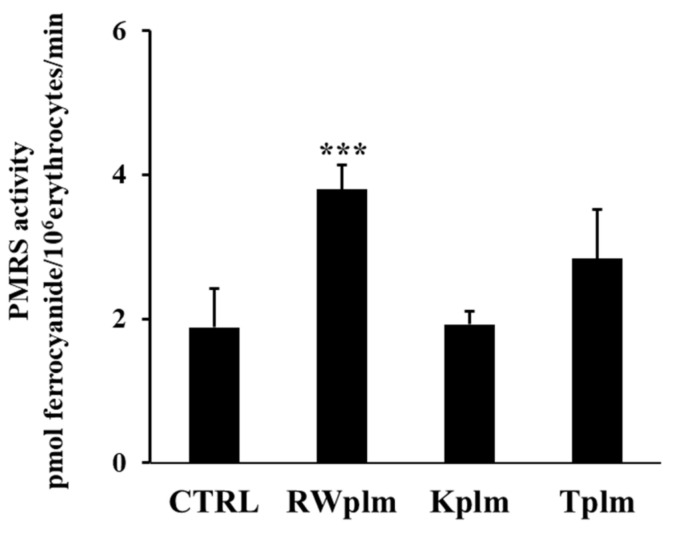
PMRS activity of erythrocytes treated with RWplm, Kplm and Tplm. Erythrocytes from volunteers (*n* = 5) were incubated with RWplm, Kplm and Tplm (0.12 mg/mL, *w*/*V*) for 10 min. Results were expressed as pmol ferrocyanide/10^6^ erythrocytes/min as reported in “Materials and Methods”. Bar graphs represent the mean of 5 independent determinations, each performed in duplicate, ± SE. Symbols (*) in the graph indicate statistical significance: *** *p* < 0.001 respect to untreated cells (CTRL).

Data reported in Figure 5 show that Kplm and Tplm (0.12 mg/mL, *w/v*) were unable to significantly increase PMRS activity compared to untreated erythrocytes (CTRL), underlining that RWplm possessed a specific capacity to activate PMRS compared to the polymers from other beverages.

**Figure 6:**
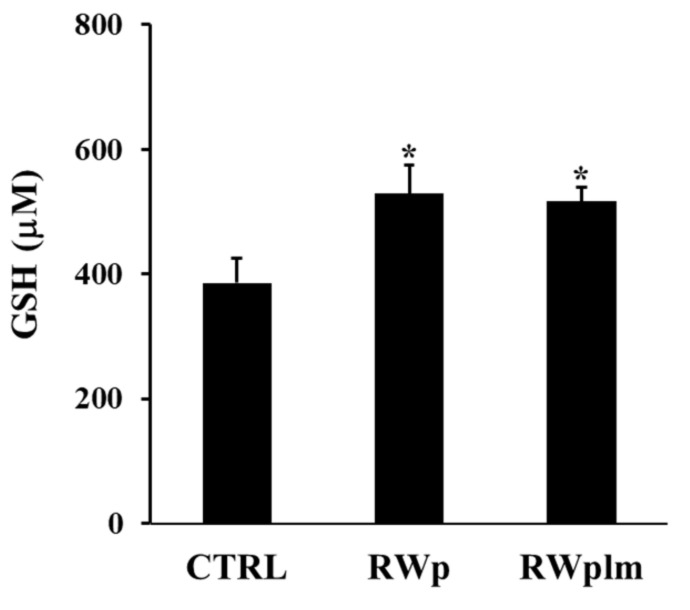
GSH intracellular concentration in erythrocytes. Erythrocytes from volunteers (*n* = 5) were incubated with the Scheme 73. and 27 μg/mL GAE, respectively) for 10 min. Results were expressed in terms of μM GSH as reported in “Materials and Methods”. Bar graphs represent the mean of 5 independent determinations, each performed in duplicate, ± SE. Symbols (*) in the graph indicate statistical significance: * *p* < 0.05 respect to untreated (CTRL).

Moreover, the ability of RWplm to increase GSH intracellular concentration was tested after treatment of erythrocytes with the same volumes of RWp and RWplm (corresponding to 73 and 27 μg/mL GAE, respectively). As showed in Figure 6, GSH was significantly increased by both RWp and RWplm treatments of comparable values (27 and 25%, respectively, with respect to the untreated control). In this case, no significant difference was measured between RWp and RWplm, confirming the contribution of both preparations to the biological activity of RWp.

## 4. Discussion

The present work indicates the existence of a novel mechanism triggered by RWp in strengthening erythrocyte antioxidant defenses through the activation of PMRS. Considering the biological role of oxygen carriers, PMRS represents the first protection mechanism from oxidative stress to neutralize plasma free radicals in erythrocytes. The activity of PMRS can be modulated by compounds capable of supplying electrons to the transport chain, contributing to the compensatory mechanism to balance oxidative stress. It is generally accepted that several polyphenolic compounds, such as resveratrol, myricetin, and quercetin, can potentiate cellular antioxidant systems, including PMRS [45,46,47]. For the presence of hydroxyl groups in their chemical structure, polyphenols appeared as excellent candidates to increase PMRS, acting as intracellular electron donors to this system.

**Figure 7:**
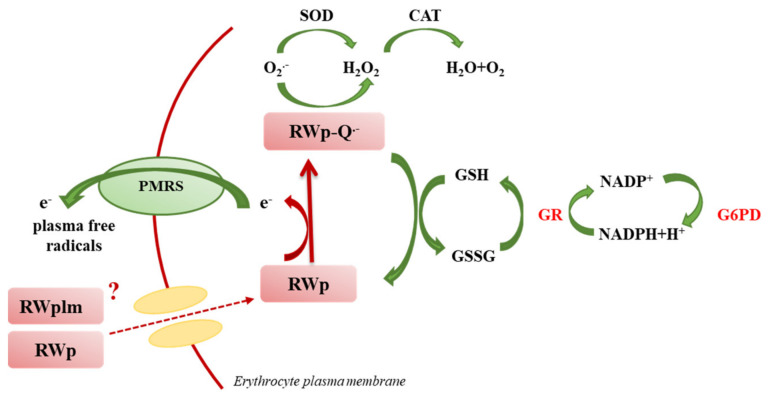
Original graphical scheme representing the proposed mechanisms underlying the protective effect of RWp (see text for description).

We previously reported the ability of RWp (73 μg/mL GAE) to increase PMRS activity in human erythrocytes [26]. The present data suggest that RWp increase ROS concentration generating a pro-oxidant effect responsible for the induction of adaptive stress-response that can protect erythrocytes against a variety of adverse conditions. Changes in ROS levels, such as those induced by RWp (Figure 1), finely regulate the redox environment through a strong network that leads to an increase in antioxidant systems. To provide a possible explanation of the pro-oxidant effect of RWp, we hypothesized that, once inside the cell, RWp components supply electrons to PMRS, resulting in their subsequent self-oxidation and forming semiquinone radical species (RWp-Q^•−^) (Figure 7). Evidence exists on the possibility that polyphenols could behave as pro-oxidants under specific conditions [48,49]. This process is not totally new, in fact, when polyphenols lose an electron or act as reducing agents, they become radicals and their oxidized intermediates can react with transition metal ions forming oxidation products, such as semiquinones and quinones, promoting the chain propagation of reactive species [50]. The semiquinone radicals are highly unstable and undergo further oxidations, producing more stable quinones [51]. Auto-oxidation of apigenin, naringenin, and naringin produces superoxide radicals, also occurs in presence of transition metals [52,53]. As an example, the pro-oxidant activity of flavonoids is supposed to be strictly correlated to the total number of hydroxyl groups, the presence of the double bond in 2–3 position of the C ring, and of the hydroxyl groups in 3′ and 5′ positions of the B ring, which may increase the hydroxyl radical production in the Fenton reaction [54].

The pro-oxidant activity of polyphenols can be beneficial in the cellular environment since the induction of moderate oxidative stress stimulates the activation of cell defense systems [55]. Indeed, important literature evidence exists that low concentrations of ROS, like those promoted by RWp, are involved in normal cellular functions, as well as disease prevention [56,57]. The role of polyphenols inducing adaptive stress-response pathways that can protect against oxidative damage has been previously reported in other cellular models [58,59]. Furthermore, it should be considered that low concentrations of free radicals are associated with the functionality of several redox membrane enzymes, including PMRS, mainly membrane-residing NADPH oxidases, which convert O_2_ into superoxide (O_2_^•-^). This could also explain the activation of SOD (Figure 3a) which catalyzes the dismutation of superoxide (O_2_^•-^) into H_2_O_2_ and O_2_ and CAT (Figure 3b) to neutralize H_2_O_2_.

To restore redox homeostasis, following the increase in ROS concentration, GSH is the most important non-enzymatic antioxidant defense system, which reacts with intracellular radical intermediates, including RWp-Q^•−^, and is converted into GSSG (Figure 7). Shifts in the cellular GSH redox state, like those triggered by RWp, can be restored by the activity of GR and G6PD enzymes (Figure 7). Based on these observations, the strengthening of erythrocyte antioxidant systems, including GSH and GSH-dependent enzymes, exerted by RWp can be seen as a protective mechanism against the oxidative insult, through the induction of an adaptive response following a slight but significant ROS increase, as we have summarized in the scheme in Figure 7.

Among the several classes of polyphenols, we previously demonstrated that polyphenols such as gallic acid, resveratrol, catechin and quercetin, commonly known as PMRS activators, were unable to activate PMRS at the concentrations present in RW [26]. Here, we evaluated the activity of RWplm obtaining an initial indication that these compounds could activate antioxidant systems, including PMRS and GSH. Indeed, when we separated RWplm from RWp through dialysis, we observed a loss of total mass of RWp of about 92%, but the effect on PMRS remains unmodified with respect to RWp (Figure 4), suggesting a key role of RWplm in modulating PMRS activity. Hence, we hypothesize that RWplm can donate electrons to PMRS generating an electron flux able to increase intracellular ROS. Based on this assumption, we suggest that RWplm components behave as pro-oxidants, triggering an adaptive cellular response in the erythrocyte (Figure 7). 

An important aspect regards the issue of the cellular uptake of these high molecular weight compounds since data present in literature must be carefully considered. It has been shown that several polyphenols and metabolites, including δ-(3,4-dihydroxy-phenyl)-γ-valerolactone, can cross cell membrane by passive binding through facilitated transport via GLUT-1 transporter [60]. According to this theory, we cannot exclude that RWplm can pass the erythrocyte membrane via specific transporters or interfere with specific components of the PMRS complex.

Further studies will be devoted to the characterization of specific components of RWplm, and analyze their interaction with the erythrocyte membrane to assess the complexity of their biological response.

Finally, we do not exclude the possibility that the ability of RWp to increase the PMRS activity and protect erythrocytes from oxidative stress occurs via different mechanisms including the combined action of several compounds acting synergistically at low concentrations, a hypothesis currently under investigation.

## Figures and Tables

**Table 1:** Summary of RWp biological activity in erythrocytes [26].

Hemolysis	PMRS	GSH
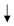	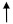	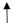

**Table 2:** ALR and MetHbR activities after treatment with RWp in erythrocytes.

Enzyme	CTRL	RWp
ALR	6508.79 ± 716.13	5600.24 ± 1214.89
MetHbR	12183.50 ± 1232.61	11587.73 ± 1181.67

Erythrocytes isolated from volunteers (*n* = 5) were incubated with RWp (73 μg/mL GAE) for 10 min.

## Data Availability

The data presented in this study are available on request from the corresponding author.
